# Spatial mapping of functional pelvic bone marrow using FLT PET

**DOI:** 10.1120/jacmp.v15i4.4780

**Published:** 2014-07-08

**Authors:** Sarah M. McGuire, Yusuf Menda, Laura L. Boles Ponto, Brandie Gross, Mindi TenNapel, Brian J. Smith, John E. Bayouth

**Affiliations:** ^1^ Department of Radiation Oncology University of Iowa Hospitals and Clinics Iowa City IA USA; ^2^ Department of Radiology University of Iowa Hospitals and Clinics Iowa City IA USA; ^3^ Department of Biostatistics University of Iowa Hospitals and Clinics Iowa City IA USA; ^4^ Department of Human Oncology University of Wisconsin School of Medicine and Public Health Madison WI USA

**Keywords:** pelvic cancer, FLT PET, bone marrow

## Abstract

The purpose of this study was to determine the ability of regions identified with bony landmarks on CT imaging to accurately represent active bone marrow when compared to FLT PET imaging. These surrogate regions could then be used to create a bone marrow sparing radiation therapy plan when FLT PET imaging is not available. Whole body (WB) FLT PET images were obtained of 18 subjects prior to chemoradiation therapy. The FLT image of each subject was registered to a CT image acquired for that subject to obtain anatomic information of the pelvis. Seventeen regions were identified based on features of the pelvic bones, sacrum, and femoral heads. The probability of FLT uptake being located in each of 17 different CT‐based regions of the bony pelvis was calculated using Tukey's multiple comparison test. Statistical analysis of FLT uptake in the pelvis indicated four distinct groups within the 17 regions that had similar levels of activity. Regions located in the central part of the pelvis, including the superior part of the sacrum, the inner halves of the iliac crests, and the L5 vertebral body, had greater FLT uptake than those in the peripheral regions (p‐value < 0.05). We have developed a method to use CT‐defined pelvic bone regions to represent FLT PET‐identified functional bone marrow. Individual regions that have a statistically significant probability of containing functional bone marrow can be used as avoidance regions to reduce radiation dose to functional bone marrow in radiation therapy planning. However, because likely active bone marrow regions and pelvic targets typically overlap, patient‐specific spatial detail may be advantageous in IMRT planning scenarios and may best be provided using FLT PET imaging.

PACS number: 87.57.uk

## I. INTRODUCTION

Overall survival of pelvic cancer patients depends on control of systemic disease. If local radiation therapy depletes bone marrow function to such an extent that systemic therapies must be withheld, chances of metastatic failure increase significantly. This may be more significant for this group of patients because approximately one‐third of hematopoietically active adult bone marrow is located in the pelvic region.^1^, ^2^ Strategies to minimize toxicities would benefit a range of pelvic cancer patients including gynecologic, anal, rectal, and prostate patients. New chemoradiation combinations improve outcomes for these disease sites, but come at the cost of higher levels of hematologic toxicity.^3^, ^4^, ^5^, ^6^, ^7^, ^8^, ^9^, ^10^ Hematologic toxicity is a major contributor to missed chemotherapy cycles in cervical cancer patients and has also been shown to be independently predictive of worse progression‐free and overall survival for these patients.^(11)^ Little data are available as to what extent hematologic toxicity contributes to shortened or suspended chemotherapy administrations in other pelvic disease sites. However, limiting treatment‐related toxicities for all pelvic cancer patients increases the likelihood that patients could complete the prescribed course of therapy and return to their nominal activities. In addition, reduced toxicities offer the potential option of dose escalation, when warranted, to improve therapy outcomes.

To successfully limit hematologic toxicities for pelvic cancers, strategies must be developed to limit radiation dose to the highly proliferative compartments of the pelvic bone marrow. However, the complex structure of the pelvis makes it difficult to assess the efficacy of radiation therapy (RT) planning strategies to avoid areas critical to hematopoiesis.^12^, ^13^ Current strategies that rely on CT data alone used intensity‐modulated radiation therapy (IMRT) to spare peripheral portions of the pelvic bones^12^, ^13^, ^14^ or dose to the pelvic bones as a predictor of hematologic toxicity as a surrogate for dose to active bone marrow.^15^, ^16^, ^17^ Uptake of [^18^F] fluorothymidine imaged with positron emission tomography (FLT PET) can be an accurate and sensitive tool for identifying actively proliferating bone marrow in order to incorporate these areas as avoidance regions in radiation therapy plans.^18^, ^19^ However, FLT is not yet an FDA‐approved radiopharmaceutical and, therefore, is not readily available at most institutions. The combination of FLT PET and CT imaging may add to current CT‐based techniques and expand the potential benefits of bone marrow sparing radiation therapy plans to a larger group of patients. An alternative CT‐based method could be to create probabilistic maps of marrow function based strictly on bony anatomy (e.g., radiation therapy simulation CT). This anatomic strategy would subdivide the bony pelvis into regions that have a greater probability of containing active bone marrow based on FLT uptake. This method would offer the possibility of locating and sparing active bone marrow using the radiation therapy CT image alone, negating the need for further procedures or diagnostic radiation exposure. The purpose of this study was to determine the ability of regions identified using CT imaging to accurately predict the location of active bone marrow when compared to FLT PET for the majority of patients. These surrogate regions could then be incorporated into the IMRT optimization process to create a bone marrow sparing radiation therapy plan.

## II. MATERIALS AND METHODS

### A. FLT PET imaging to quantify cell proliferation in the pelvic bone marrow

FLT is a thymidine analog which is retained in the cell through phosphorylation by thymidine kinase 1 (${\text{TK}}_{1}$).^(20)^ ${\text{TK}}_{1}$ is a key enzyme in the synthesis of DNA and shows markedly enhanced activity during the S‐phase of the cell cycle. FLT uptake in tissue is considered a marker of active cellular proliferation and DNA replication, although FLT is not incorporated into the DNA.^(21)^

FLT PET datasets were obtained within 30 days prior to initiation of chemoradiation therapy from subjects enrolled in either a previous study of head and neck cancer patients (NCT00721799) or cervical cancer patients (NCT01075412) at our institution, and analyzed retrospectively. Acquisition of FLT PET images was previously described by Menda et al.^(22)^ Briefly, FLT was produced based on the method described by Machulla et al.^(23)^ Images of the head and neck cancer subjects were acquired on an ECAT EXACT HR+ PET scanner (Siemens Medical Solutions USA, Inc. Knoxville, TN) operated in the three‐dimensional mode after intravenous infusion (over 2 min) of FLT (2.6 MBq/kg (0.07 mCi/kg), maximum dose=185 MBq(5mCi)). Whole‐body (WB) images from the base of skull to mid‐femur were obtained at 74±7 min postinfusion. Data from 12 subjects, who had both WB FLT PET images and WB FDG PET images with an attenuation correction CT (AC CT) extending to mid‐femur, were used for this study. Attenuation correction was used for both PET images, but was CT‐based only for the WB FDG PET image. As a result, the AC CT was used to provide anatomic information, since no other CT information of this region was acquired. Images of six cervical cancer subjects were obtained on a PET/CT scanner (Siemens Biograph 40 PET/CT Scanner) from base of brain to the proximal thighs (3 min/bed position) at approximately 60–70 min postadministration of FLT (2.6 MBq/kg (0.07 mCi/kg), maximum dose=185 MBq(5mCi)). A simulation CT of the pelvis was also acquired for these subjects as part of the radiation therapy planning process. This simulation CT was used to provide anatomic information for these subjects. All CT and FLT PET images were transferred to the Pinnacle Treatment Planning system v9.2 (Philips Medical Systems, Fitchburg, WI) for analysis. Of the 18 total subjects from both the head and neck and cervical cancer studies, 11 were male and 7 were female. The mean age for all subjects was 53 years, with a range of 39 to 71 years.

### B. Identification of bone regions within the pelvis

**Figure 1:**
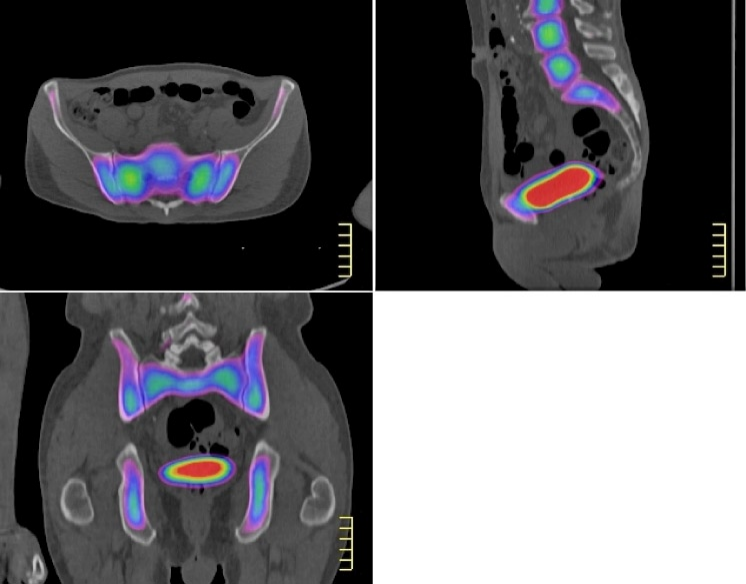
Axial, sagittal, and coronal views of the registered FLT PET and CT images for one subject. Color wash represents increasing FLT uptake from purple to red and blue approximately equal to SUV 2.

FLT PET images for 18 subjects were registered to the available CT on the treatment planning system. The FLT PET image from the head and neck cancer study was registered to the FDG PET AC CT manually focused in the pelvic region. The FLT PET image from the cervical cancer study was registered to the simulation CT by utilizing the transformation from the registration of its AC CT to the simulation CT which was also focused in the pelvic region. A coregistered FLT PET and corresponding CT image for one subject is shown in Fig. 1.

**Figure 2:**
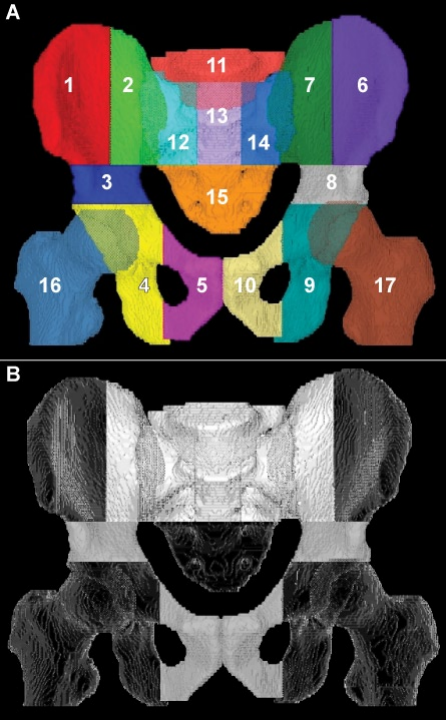
The 17 bone regions (a) identified on one subject's pelvis; the bone regions (b) grouped by similarity in FLT uptake. The subject average SUV mean is largest in the group identified in white. The group identified in grey has the second largest average SUV mean. The group identified in black has the smallest average SUV mean.

The bony pelvis for each subject was divided into 17 regions using bony landmarks identified on each subject's CT image. Figure 2(a) shows these regions for one subject. The extent of the bony pelvis was defined by the superior edge of the L5 vertebral body to the level of the inferior edge of the ischial tuberosity. Regions 1, 2, 6, and 7 were defined by the iliac crests, with an inferior border defined by the inferior edge of the S‐I joint and separated by a line that cuts the anterior and posterior regions at midacetabulum and the pelvic iliac narrowing. Regions 3 and 8 are defined from the inferior edge of the S‐I joint to the superior edge of the femoral head. Regions 4, 5, 9, and 10 are defined from the superior edge of the femoral head through the ischium separated by the line of the obturator foramen. Regions 16 and 17 were defined by the femoral heads to the inferior edge of the ischial tuberosity. Region 11 was defined by the L5 vertebral body. Regions 12, 13, and 14 were defined by the superior portion of the sacrum to the inferior edge of the S‐I joint and separated into three parts by the sacral foramina. Finally, region 15 was defined by the region inferior to the S‐I joint to the inferior edge of the sacrum.

### C. Statistical analysis of FLT PET uptake in bone regions

First, mean FLT standard uptake value (SUV) in each region was obtained by dividing the mean voxel value calculated in the treatment planning system by the voxel value/SUV calculated based on injection activity, body weight, and time for each subject. Statistical testing was then performed using SAS software (Cary, NC). A Tukey's multiple comparison test was used to compare FLT SUV means calculated in the individual CT identified pelvic regions to one another. This test was used to determine if the SUV means differed between regions while controlling for multiple tests. An individual region was compared against all other regions for a particular subject and against that region across subjects. P‐values were used to determine which regions had similar SUV means. Pairs with an adjusted p−value<0.05 were considered to be significantly different and pairs with a p−value>0.5 were considered to be similar. The similarity threshold was arbitrarily chosen.

## III. RESULTS

Statistical analysis of FLT uptake in the 17 bone regions comparing each region to all the other regions for each subject indicated a pattern in FLT uptake and provides a method to predict the most likely location of significant uptake in the bony pelvis. This analysis resulted in the following groups of bone regions based on similarity (p−value>0.5): Group 1 contains regions 2, 7, 11, 12, 13, and 14; Group 2 contains regions 3, 5, 8, and 10; Group 3 contains regions 1, 4, 6, and 9; and Group 4 contains regions 15, 16, and 17. Region 15 had the lowest mean SUV of all regions, but was grouped with regions 16 and 17 since all three are significantly lower than those regions in Group 3 and are not significantly different (p−value>0.05).

Group 1 consisted of the upper central region of the pelvis including the superior portion of the sacrum, the inner halves of the iliac crests, and the L5 vertebral body. Group 2 consisted of the lower central region of pelvis including the central halves of the ischial tuberosities. Groups 3 and 4 consisted of the more peripheral regions of the pelvis including the outer rims of the iliac crests, the outer halves of the ischial tuberosities, and the femoral heads, with the exception being region 15 containing the sacrum inferior to the S‐I joint. The mean SUV for each region was averaged across all 18 patients. Table 1 shows the average FLT SUV means ranked in order from highest to lowest. Group 1 contained the six regions with the largest average SUV means which ranged from 3.3 to 3.7. Group 2 contained the four regions with the next largest average SUV means ranging from 2.6 to 2.9. Group 3 contained average SUV means ranging from 2.2 to 2.3, and Group 4 contained average SUV means ranging from 1.2 to 1.7. This indicates that the majority of FLT uptake occurs in the central region of the pelvis, excluding the sacrum below the S‐I joint. If region groups with an average SUV mean less than 2.3 can be considered to contain insignificant amounts of active bone marrow, then Groups 3 and 4 could be combined into a broader group that would have the least amount of active bone marrow. Figure 2(b) shows the location of Group 1 (white), Group 2 (grey), and Groups 3 and 4 (black) for one subject.

**Table 1:** Pelvic bone regions ranked by mean FLT SUV averaged over 18 subjects

*Rank*	*Region*	*Average FLT SUV*	*Standard Deviation*
*Group 1*			
1	11	3.71	1.00
2	12	3.65	0.93
3	14	3.57	0.90
4	2	3.52	0.87
5	7	3.49	0.98
6	13	3.28	0.82
*Group 2*			
7	3	2.93	1.08
8	8	2.92	1.08
9	5	2.69	0.65
10	10	2.64	0.59
*Group 3*			
11	9	2.26	0.85
12	1	2.24	0.63
13	4	2.23	0.84
14	6	2.16	0.57
*Group 4*			
15	16	1.69	1.10
16	17	1.68	1.11
17	15	1.23	0.56

## IV. DISCUSSION

The utility of FLT PET to identify active bone marrow throughout the body has been demonstrated for the purposes of identifying hematologic disorders,^(18)^ but a population‐based analysis of normal active bone marrow could have potential utility in radiation treatment planning. Statistical analysis using a region‐by‐region comparison indicated four multiregion groups whose regions were statistically the same in regard to FLT SUV mean. These groups also corresponded in rank order of average FLT SUV mean, with Group 1 having the largest average FLT SUV mean, followed by Groups 2, 3, and 4. The location of these groups indicates that the upper central region of the pelvis has the greatest likelihood of containing more FLT uptake and, therefore, active bone marrow, than the other regions of the pelvis.

Grouping regions of bone that can be readily identified using only a radiation therapy simulation CT offers the potential for clinically useful bone marrow sparing without the need for additional imaging studies. FLT PET offers a more precise and patient‐specific methodology of locating active bone marrow in the pelvis, but FLT is not yet a readily available FDA‐approved PET radiopharmaceutical. CT‐defined bone regions, validated with FLT PET uptake analysis, offer a more readily available method to locate the most probable location of active bone marrow to use in intensity‐modulated radiation therapy (IMRT) planning in order to reduce hematologic toxicity in patients with pelvic cancers. These data indicate that regions can be prioritized into three major groups based on marrow FLT uptake. The most central regions have the greatest likelihood for containing active bone marrow, and the more peripheral regions have the greatest likelihood for containing relatively less active bone marrow.

A CT‐defined bone region‐based approach could alleviate the problem of the limited availability of FLT, and the time and expense of additional imaging for the sole purpose of identifying bone marrow. However, the location of the active bone marrow indicated by our results presents another problem in a radiation therapy planning scenario. Pelvic tumors are centrally located and would, therefore, be in close proximity to the pelvic bone regions with the largest amount of active marrow. In a previous study evaluating the ability to reduce planned radiation dose to FLT‐identified active bone marrow, the volumes of active bone marrow receiving 10 and 20 Gy could be reduced by 10%−30% without significantly compromising target constraints or other organ at risk (OAR) objectives.^(19)^ Bone marrow dose reductions were calculated based on the entire volume of FLT SUV 2, 3, and 4 within the pelvis. Therefore, it is difficult to know where the dose reduction occurred within the volume. CT‐defined bone regions and groups representing active bone marrow allow for guidance volumes for IMRT optimization based on bone alone, but there will be locations within those regions that do not contain as much active bone marrow. It may be that those regions with smaller volumes of active bone marrow contribute most to the possible total active bone marrow dose reduction. This problem may be somewhat alleviated by developing dose objectives based on analysis using FLT uptake change, which could then be translated to a bone region model. Still, there would be uncertainties in uptake based on interpatient variability that could not be predicted and taken into account.

Other methods exist for identifying active bone marrow, such as sulfur colloid SPECT^(24)^ imaging and a combination of fat quantification MR imaging and FDG PET imaging.^(25)^ Both of these methods are more readily available than FLT PET imaging. However, sulfur colloid SPECT imaging lacks the quantitative abilities of PET imaging, and the combination of fat quantification MR and FDG PET regions adds an extra degree of complexity to the identification process with the need for two sets of images. Fat quantification protocols are not necessarily standardized or readily available on many clinical MR scanners. Both of these methods also require extra imaging time and radiation dose to the patient, similar to FLT PET imaging. Unlike FLT PET, these imaging techniques are less straightforward in identifying proliferating bone marrow than the DNA synthesis that uptake by FLT into cells represents. No correlation has been reported between FLT PET and either sulfur colloid SPECT imaging or fat quantification MR and FDG PET imaging. Correlation of both sulfur colloid SPECT and the combination of MR and FDG PET imaging to FLT PET imaging could help to establish the reliability of each method, and provide other tools that could be used to identify pelvic bone marrow for patient‐specific bone marrow sparing radiation therapy planning.

## V. CONCLUSIONS

Using only the primary planning CT for identifying active pelvic bone marrow to incorporate into IMRT planning is potentially feasible based on our statistical analysis of FLT uptake in 18 subjects. FLT PET imaging provides better spatial sensitivity for identifying bone marrow, but is limited in that it is not readily available in a clinical setting, and requires extra imaging time and radiation exposure. The use of bone region surrogates identified on a simulation CT offers a better probability of sparing active bone marrow than using the entire pelvic bone, but may still be limited by the overlap of pelvic radiation therapy targets.

## ACKNOWLEDGMENTS

The authors acknowledge and thank the clinical trials research team responsible for this study, including K. Bodeker, J. Hershberger, S. Vollstedt, and J. Koeppel. This research was funded by the National Cancer Institute (1R21 CA130281‐01 and 3P30CA086862).
